# The Metabolic Landscape of Lung Cancer: New Insights in a Disturbed Glucose Metabolism

**DOI:** 10.3389/fonc.2019.01215

**Published:** 2019-11-15

**Authors:** Karolien Vanhove, Geert-Jan Graulus, Liesbet Mesotten, Michiel Thomeer, Elien Derveaux, Jean-Paul Noben, Wanda Guedens, Peter Adriaensens

**Affiliations:** ^1^UHasselt, Faculty of Medicine and Life Sciences, LCRC, Diepenbeek, Belgium; ^2^Department of Respiratory Medicine, Algemeen Ziekenhuis Vesalius, Tongeren, Belgium; ^3^Biomolecule Design Group, Institute for Materials Research, Hasselt University, Diepenbeek, Belgium; ^4^Department of Nuclear Medicine, Ziekenhuis Oost Limburg, Genk, Belgium; ^5^Department of Respiratory Medicine, Ziekenhuis Oost Limburg, Genk, Belgium; ^6^Biomedical Research Institute, Hasselt University, Diepenbeek, Belgium; ^7^Applied and Analytical Chemistry, Institute for Materials Research, Hasselt University, Diepenbeek, Belgium

**Keywords:** lung cancer, glucose, metabolism, genetic alterations, targeting metabolism

## Abstract

Metabolism encompasses the biochemical processes that allow healthy cells to keep energy, redox balance and building blocks required for cell development, survival, and proliferation steady. Malignant cells are well-documented to reprogram their metabolism and energy production networks to support rapid proliferation and survival in harsh conditions via mutations in oncogenes and inactivation of tumor suppressor genes. Despite the histologic and genetic heterogeneity of tumors, a common set of metabolic pathways sustain the high proliferation rates observed in cancer cells. This review with a focus on lung cancer covers several fundamental principles of the disturbed glucose metabolism, such as the “Warburg” effect, the importance of the glycolysis and its branching pathways, the unanticipated gluconeogenesis and mitochondrial metabolism. Furthermore, we highlight our current understanding of the disturbed glucose metabolism and how this might result in the development of new treatments.

## Introduction

The metabolic alterations of cancer cells, that distinguish them from healthy cells, are recognized as one of the ten hallmarks of cancer. An altered metabolism helps cancer cells to sustain high proliferative rates even in a hostile environment resulting from a poor vascularization, which limits the supply of oxygen (O_2_) and essential nutrients (1).

In the 1920s, Otto Warburg postulated that tumor cells consume glucose and excrete lactate at a significantly higher rate compared to healthy resting cells (2). Even in normoxic conditions, proliferating cells, such as cancer cells, rely on fermentation, i.e., glycolysis resulting in the generation of lactate via fermentation of pyruvate. The increased reduction of pyruvate to lactate and the passage of glycolytic intermediates into diverse biosynthetic pathways reduces the available concentration of pyruvate to form acetyl-CoA and to drive the tricarboxylic acid (TCA) cycle. In contrast with the original hypothesis of Warburg, the mitochondrial metabolism remains vital for both the production of ATP and the supply of biosynthetic intermediates (3). The TCA cycle or Krebs cycle is a mitochondrial pathway where acetyl-CoA undergoes a condensation reaction with oxaloacetate (OAA) to form carbon dioxide (CO_2_). In successive oxidation reactions, the coenzymes NAD^+^ and FAD are reduced and subsequently used to drive the generation of the majority of ATP by oxidative phosphorylation (OXPHOS). Although the Warburg effect is often found in malignant tumors, OXPHOS still has a significant contribution to the energy supply of at least some cancers (4, 5). Furthermore, metabolic intermediates are deviated toward biosynthetic processes operational in growing and proliferating malignant cells. To compensate for the ongoing drainage of TCA cycle metabolites into anabolic pathways, glutamine is often used in cancer cells as a carbon source to replenish TCA cycle intermediates (6, 7).

In this review, we focus on the altered glucose metabolism in lung cancer cells. As lung cancer is by far the leading cause of cancer death with limited curative treatment options, detailed understanding of the dysregulated glucose metabolism and its associated signaling pathways may help us to design more efficient treatment regimens (8, 9).

## Glycolysis: ATP and Building Blocks

**Figure 1:**
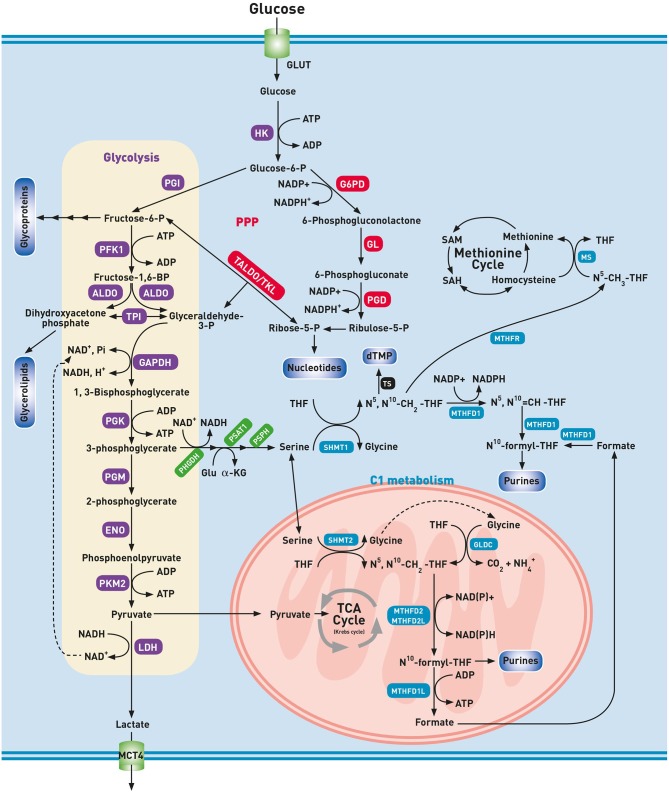
Glycolysis and biosynthetic pathways emanating from glycolysis. ALDO, aldolase; dTMP, deoxythymidine monophosphate; ENO, enolase; GAPDH, glyceraldehyde-3-phosphate dehydrogenase; GL, gluconolactonase; Glu, glutamine; GLUT, glucose transporter; G6PD, glucose-6-phosphate 1-dehydrogenase; GLDC, glycine cleavage system P protein; HK, hexokinase; LDH, lactate dehydrogenase; MCT4, monocarboxylate transporter 4; MS, methionine synthase; MTHFD, methylenetetrahydrofolate dehydrogenase; MTHFR, methylenetetrahydrofolate reductase; ${\text{NH}}_{4}^{+}$, ammonia; N^5^-CH_3_-THF, methyl-tetrahydrofolate; N^5^N^10^-CH_2_-THF, methylene-tetrahydrofolate; N^5^N^10^-CH=THF, methenyl-tetrahydrofolate; N^10^-formyl-THF, formyl-tetrahydrofolate; PFK1, phosphofructokinase 1; PGM, phosphoglycerate mutase; PGD, 6-phosphogluconate dehydrogenase; PGI, phosphoglucoisomerase; PGK, phosphoglycerate kinase; PHGDH, phosphoglycerate dehydrogenase; PKM2, pyruvate kinase M2; PPP, pentose phosphate pathway; PSAT1, phosphoserine aminotransferase 1; PSPH, phosphoserine phosphatase; SAH, S-adenosylhomocysteine; SAM, S-adenosylmethionine; SHMT, serine hydroxyl-methyltransferase; TALDO, transaldolase; THF, tetrahydrofolate; TKL, transketolase; TPI, triose phosphate isomerase; TS, thymidylate synthetase. Glycolysis (purple), One-carbon metabolism (blue), PPP (red), Serine biosynthesis (green), Other pathways (black).

During glycolysis, each molecule of glucose is broken down in ten steps to two molecules of pyruvate resulting in a net gain of two molecules of NADH and two ATP. In the presence of O_2_, healthy cells further oxidize pyruvate to CO_2_ through the mitochondrial located oxidative pathways, i.e., the TCA cycle and OXPHOS. Starting from one molecule of glucose, the combined action of the pathways mentioned above, generally known as aerobic respiration, results in the production of water as well as at least 32 ATP molecules. Under anaerobic conditions, pyruvate is reduced to lactate by lactate dehydrogenase (LDH), and lactate is secreted in the extracellular space by monocarboxylate transporters (MCT). Unlike healthy cells, lung cancer cells metabolize glucose via lactic acid fermentation even in the presence of sufficient O_2_. This metabolic condition received a plethora of names, such as aerobic fermentation, aerobic glycolysis or Warburg effect (10). Otto Warburg observed that cancer cells generate ATP through a non-oxidative pathway, i.e., glycolysis with the generation of lactic acid, even in normoxic conditions, and attributed this to mitochondrial dysfunction. To emphasize this process in the presence of O_2_, the historical concept of Warburg has led to the misleading term “aerobic glycolysis.” In our opinion, the term “aerobic fermentation” as coined by Warburg himself as “a property of all growing cancer cells” seems more appropriate to denote the fermentation in the presence of O_2_ (2). Aerobic fermentation is nowadays seen as a hallmark of rapid cell proliferation even in a non-cancerous context (11). As compared to aerobic respiration, (an)aerobic fermentation produces a 16-fold lower amount of ATP per glucose consumed, making it an inefficient way of generating ATP. However, under the non-limiting supply of glucose, a ~15 times higher glycolytic flux can be reached as compared to TCA cycle flux and consequently, a drastic increase in ATP production rate in aerobic fermentation (12). After the phosphorylation of glucose by hexokinase (HK), glucose-6-phosphate can no longer leave the cell. This combined activity of glucose uptake and its subsequent phosphorylation forms the basis for Positron Emission Tomography (PET) imaging in which an injected radioactive glucose analog (^18^F-FDG) is detected in higher concentrations in lung cancer tissue than in healthy tissues (13, 14). Currently, metabolic imaging with ^18^F-FDG-PET is regarded as a standard of care in the management of lung cancer (15, 16). The high intracellular concentrations of glucose-6-phosphate (glucose-6-P) are indispensable to maintain high glycolytic activity, and thus upregulation of HK and the glucose transporter GLUT are essential. The upregulation of the isoform GLUT1 and the relation with the uptake of ^18^F-FDG have been demonstrated in lung cancer tissue, as well as overexpression of the HK2 isoform (17, 18). Glucose-6-phosphate has to continue along the glycolytic pathway to result in the final product pyruvate in aerobic, or lactate in anaerobic conditions (Figure 1). The upregulation of almost all glycolytic enzymes has been demonstrated, including HK2 and phosphofructokinase 1 (PFK1) that catalyzes the committed step in glycolysis namely, the phosphorylation of fructose-6-phosphate into fructose-1,6-bisphosphate (19). Fructose-1,6-bisphosphate is subsequently converted into dihydroxyacetone phosphate (DHAP) and glyceraldehyde-3-phosphate (GAP) by aldolase (ALDO). In contrast with GAP, DHAP is not on the direct pathway of glycolysis. To prevent loss of this three-carbon fragment, and thus ATP, DHAP is isomerized to GAP by triose-phosphate isomerase (TPI). The resulting GAP is oxidized by glyceraldehyde-3-phosphate dehydrogenase (GAPDH) into 1,3-bisphosphoglycerate (1,3-BPG). As this reaction is at the expense of NAD^+^, the NADH formed by this reaction must be continuously re-oxidized to NAD^+^ for glycolysis to continue. Hence, the fate of lactate production from pyruvate finds its rationale in this recycling process. The importance of this reaction is demonstrated by a decreased survival and proliferation of lung cancer cells during the inhibition of LDH (20). Excretion of lactate through MCT4 transporters does not only result in the acidification of the microenvironment, but also modulates the immune cell function and promotes invasion and metastasis (21). The microenvironment in which lung cancer cells live is heterogeneous because of ineffective tumor vascularization. As a consequence, cancer cells may be subject to hypoxia and nutrient deprivation. Interestingly, swapping of lactate between hypoxic and oxygenated cells has been reported (22–24). Using MCT1 transporters, normoxic lung cancer cells can remove lactate from the microenvironment and convert it to pyruvate for further oxidation, conserving glucose for use by the hypoxic cells. In contrast with the initial hypothesis of Warburg, a majority of human cancers, including lung cancer, produces ATP through OXPHOS (25). Besides for ATP production, a high glycolytic rate is imperative to support cancer cell proliferation by supplying building blocks to duplicate the cell biomass and genome at each cell division (26). In this context, the Warburg effect or aerobic fermentation has been hypothesized to support the biosynthetic requirements of uncontrolled proliferation rather than ATP generation. The excess glycolytic carbon is deviated to multiple anabolic pathways that branch off from the glycolytic pathway (Figure 1).

A remarkable enzyme that supports the metabolism in lung cancer cells is pyruvate kinase (PK). PK catalyzes the transfer of phosphate from phosphoenolpyruvate (PEP) to ADP to produce ATP and pyruvate. PK comprises four isoenzymes (L, R, M1, and M2) derived from two genes. Cancer cells prefer expressing the PKM2 form by alternative splicing. The isoenzyme PKM2 occurs in a dimeric or tetrameric form. The tetrameric form has a high affinity to PEP and is present in normal proliferating cells. In contrast, the dimeric form is defined by a lower affinity to PEP. Lung cancer cells are characterized by expression of a dimeric form of PKM2 which implies that all glycolytic intermediates preceding PKM2 activity accumulate and are directed into biosynthetic processes, such as nucleotide-, lipid- and serine/glycine synthesis which stimulates tumor proliferation as demonstrated in Figure 1 (27–29).

## Metabolic Pathways Emanating From Glycolysis

### The Pentose Phosphate Pathway (PPP)

The PPP consists of two phases: a reversible non-oxidative phase and an irreversible oxidative phase. Overexpression and upregulation of two enzymes of the oxidative phase, i.e., glucose-6-phosphate 1-dehydrogenase (G6PD) and 6-phosphogluconate dehydrogenase (PGD), contributes to increased production of NADPH and ribose-5-phosphate in lung cancer (30). NADPH is a principal reducing agent that is employed in biosynthetic pathways, such as the synthesis of fatty acids, cholesterol and nucleotides. Furthermore, NADPH is oxidized during the reduction of oxidized glutathione (GSSG) to glutathione (GSH), which is essential for the detoxification of reactive oxygen species (ROS). To keep hypoxia-induced ROS due to aberrant vascularization in balance, reduced glutathione and thus NADPH is required (31). Ribose-5-phosphate is an essential building block of coenzymes as well as purine and pyrimidine nucleotides. In contrast with healthy cells, the non-oxidative phase of the PPP seems to be important in lung cancer cells (32–34). The glycolytic intermediates fructose-6-phosphate (fructose-6-P) and GAP are diverted toward ribose-5-phosphate production by transaldolase and transketolase (35). Transketolase-like-protein 1 (TKTL1) protein, a transketolase associated with the condition of aerobic fermentation is overexpressed in lung cancer cells resulting in a higher amount of ribose-5-phosphate (ribose-5-P) than needed for de novo synthesis of purines and pyrimidines (33, 34).

### The Hexosamine Biosynthetic Pathway (HBP)

Fructose-6-phosphate can branch off from the glycolytic pathway as a substrate in the HBP. The upregulated import of both glucose and glutamine results in an increased flux through the HBP and an increased level of its end product UDP-GlcNAc (36). UDP-GlcNAc is an essential metabolite for synthesis of many glycoconjugates, such as glycosaminoglycans, glycolipids, and glycoproteins. Lung cancer cells exhibits striking alterations in glycosylation but their complete description is out of the scope of this review, and Lemjabbar-Alaoui et al. described this extensively (37). O-GlcNAcylation, i.e., the enzymatic addition of the N-acetylglucosamine moiety of UDP-GlcNAc to the hydroxyl groups of serine and threonine residues, is of particular interest in lung cancer. As UDP-GlcNAc is the end product of the HBP, a pathway that makes direct use of glucose and glutamine inputs, the O-GlcNAcylation is modulated by nutrient availability and thereby acts as a nutrient sensor and metabolic regulator (38). The process of O-GlcNAcylation is regulated by O-GlcNAc-transferase (OGT) and its opponent O-GlcNAcase (OGA). Mi et al. demonstrated an elevated expression of OGT and an increased O-GlcNAcylation in lung cancer tissue. However, there was significant difference in OGA levels between cancer tissue and adjacent healthy tissue (39).

O-GlcNAcylation, an epigenetic modification of cellular proteins, oncogenes, and tumor suppressor genes, can significantly impact tumor growth, proliferation, invasion, and metastasis (40). For instance, the oncogene c-MYC is frequently expressed at constitutive high levels. Once activated by an extracellular tyrosine kinase, the degradation of c-MYC is regulated by phosphorylation of specific sites. Increased O-GlcNAcylation of the threonine site competes with its phosphorylation, resulting in the stabilization of c-MYC and sustained transcription of genes involved in the tumorigenesis. On the enzymatic level, O-GlcNAcylation is a modulator of several glycolytic enzymes (41). As an example, glycosylation of PFK1 is triggered under hypoxic conditions, and its inactivation redirects the flux of glucose from glycolysis to the PPP, thereby providing reducing power to, among other things, prevent ROS toxicity (42).

### The Serine–Glycine Pathway and One-Carbon Metabolism

An amount of glycolytic 3-phosphoglycerate (3-PG), is siphoned into serine and glycine metabolism, which provides carbon units for the one-carbon metabolism. Serine is incorporated into the head-groups phosphatidylserine and sphingolipids and is an abundant constituent of proteins (43). The serine biosynthesis pathway uses three subsequent enzymes to convert 3-PG into serine (Figure 1) (44). The increased expression of phosphoglycerate dehydrogenase (PHGDH) and the upregulation of both phosphoserine aminotransferase 1 (PSAT1) and phosphoserine phosphatase (PSPH) highlight the importance of the serine biosynthesis pathway in lung cancer biology (45, 46). Serine is the primary substrate for the so-called one-carbon cycle (47). The one-carbon metabolism, that includes both the folate and methionine cycles, is a complex metabolic network based on the biochemical reactions of folate components. A pivotal reaction of the folate cycle is the conversion of serine to glycine by serine hydroxyl-methyltransferase enzymes (cytosolic SHMT1 and mitochondrial SHMT2). This reaction generates glycine and N^5^,N^10^ methylenetetrahydrofolate (N^5^,N^10^-CH_2_-THF) which is the first one-carbon donor in the folate cycle. The knockdown of SHMT results in cell cycle arrest and cell death, suggesting that SHMT plays a crucial role in lung cancer (48). The cleavage of glycine into CO_2_ and ${\text{NH}}_{4}^{+}$ by a decarboxylase (GLDC) of the glycine cleavage system (GCS) likewise results in the production of N^5^,N^10^-CH_2_-THF. The GCS results in significant changes in both the glycolysis and serine/glycine metabolism of lung cancer patients, leading to changes in pyrimidine metabolism and cancer cell proliferation (46, 49, 50). Lung cancer cells can use N^5^,N^10^-CH_2_-THF in several ways: (i) as a one-carbon donor for the first step of thymidylate synthesis; (ii) as a substrate for N^5^,N^10^-CH_2_-THF dehydrogenase 1 (MTHFD1) or the mitochondrial tandem enzyme MTHFD2L/MTHFD2 to produce N^10^-formyl-THF, a one-carbon donor for purine synthesis; or (iii) by N^5^,N^10^-CH_2_-THF reductase (MTHFR) to generate N^5^-CH_3_-THF. This N^5^-CH_3_-THF donates its methyl group generating methionine and THF. This reaction couples the folate cycle with the methionine cycle and can be considered as the first reaction of the methionine cycle. When the resulting THF is converted into N^5^,N^10^-CH_2_-THF by SHMT, the folate cycle is closed. Methionine is the precursor of S-adenosylmethionine (SAM), a methyl donor that plays a role in both DNA and histone methylation. As reported by Mentch et al., intermediary metabolites and cofactors in one-carbon metabolism and SAM metabolism determine the DNA and histone methylation status (51). Promoter hypermethylation plays a significant role in cancer through transcriptional silencing of growth inhibitors, such as tumor suppressor genes. Together with the folate metabolites provided by SHMT-mediated reactions, SAM is vital in maintaining a regular methylation pattern and DNA stability in lung cancer (50–52). In contrast with genetic mutations, epigenetic modifications are reversible. For instance, DNA and histone methylation can be removed by α-ketoglutarate (α-KG) demethylases. The high uptake of glucose and glutamine in proliferative cells results in higher intracellular concentrations of α-KG. However, the glucose and glutamine addiction of malignant cells may end in regional depletion of both nutrients, and thus in a decrease of the α-KG concentration, resulting in the inhibition of demethylation (53). In contrast with this observation, where cell metabolites and enzymes modulate epigenetic phenomena, epigenetic modifications at metabolic genes, such as acylation or O-GlcNAcylation may affect cell metabolism. A detailed description of the link between metabolism and epigenetic changes is out of the scope of this review, and has been described extensively by Yu et al. (54). Summarized, it seems that epigenetic modifications and cellular metabolism interact with each other and that their relationship is reciprocal. Indeed, the enhanced aerobic glycolysis has a disruptive effect on tumor suppressor genes and oncogenes resulting in genomic instability. Loss of genes that are involved in the repair of DNA results in dysregulation of the mitochondrial energy production resulting in metabolic instability. In the theory of Davies et al. the interaction between genomic and metabolic instability enables pre-cancerous cells to obtain a malignant phenotype (55).

After donation of its methyl group, SAM becomes S-adenosylhomocysteine (SAH), which is subsequently converted to homocysteine. Finally homocysteine is either converted back to methionine resulting in a full turn of the cycle or enters the transsulfuration pathway to form cysteine. Cysteine can be incorporated into proteins or can be used in the formation of glutathione (52).

## The Role of Reactions of the Gluconeogenesis

**Figure 2:**
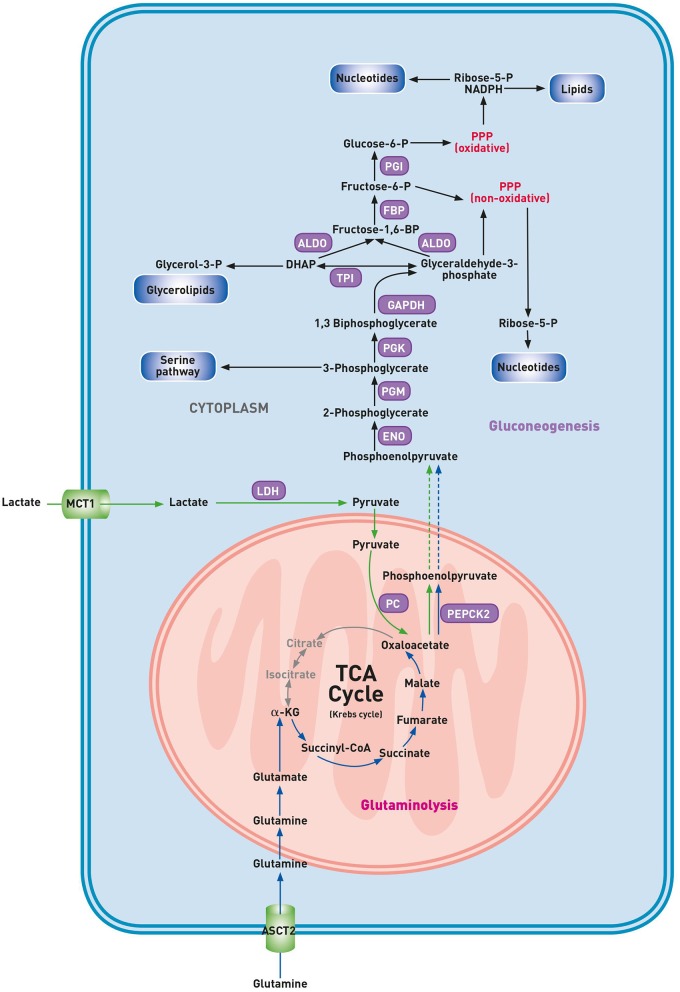
Gluconeogenesis pathway in glucose deprived lung cancer cells. α-KG, α-ketoglutarate; ALDO, aldolase; ASCT2, alanine-serine-cysteine-transporter 2; DHAP, dihydroxyacetone phosphate; ENO, enolase; FBP, fructose bisphosphatase; GAPDH, glyceraldehyde-3-phosphate dehydrogenase; LDH, lactate dehydrogenase; MCT1, monocarboxylate transporter 1; PC, pyruvate carboxylase; PEPCK2, phosphoenolpyruvate carboxykinase 2; PGM, phosphoglycerate mutase; PGI, phosphoglucoisomerase; PGK, phosphoglycerate kinase; PPP, pentose phosphate pathway; TPI, triose phosphate isomerase. Gluconeogenesis pathway (purple), lactic carbon (green arrows), glutaminolytic carbon (blue arrows).

The discovery that the activation of the gluconeogenesis pathway, until recently thought to be restricted to kidney and liver cells, also occurs in lung cancer cells, unfolds an unanticipated metabolic flexibility of cancerous cells (Figure 2) (56). Malignant cells are adapted to upregulate the glycolytic pathway at high rates. Consequently, glucose levels may drop in less perfused tumor areas. The decreased availability of glucose significantly reduces the metabolic flow via glycolysis. This reduction in glycolytic flux may result in a drop of cellular intermediates required for the biosynthesis of building blocks unless other pathways generate these glycolytic intermediates. Whereas, both the gluconeogenesis and glycolytic pathway generate identical intermediates, enhancement of either pathway could increase the supply of building blocks for cell growth. Recently, Vincent et al. described an alternative pathway in lung carcinoma cells involving phosphoenolpyruvate carboxykinase 2 (PEPCK2), a mitochondrial gluconeogenesis enzyme (57). In healthy cells, the gluconeogenesis pathway results in the production of glucose from non-carbohydrate carbon substrates. Under the condition of glucose starvation, the amino acid glutamine can maintain the TCA cycle function. Indeed, glucose-deprived malignant cells use glutamine as an anaplerotic substrate to generate α-ketoglutarate (α-KG) and subsequent TCA cycle intermediates (58). Glutamine-derived oxaloacetate is converted into PEP by mitochondrial PEPCK2, and this glutamine-derived PEP may be used for anabolic purposes (57). Indeed, conversion of PEP into 3-PG by enolase (ENO) and phosphoglyceromutase (PGM) might result in a deviation from the gluconeogenic pathway into the biosynthesis of serine, glycine, glutathione and purine nucleotides. Glutamine-derived PEP may also fuel other biosynthetic pathways that are, in normal conditions, supported by glucose, including the conversion of 1,3-BPG into glycerol for the lipid biosynthesis and utilization of GAP by the non-oxidative branch of the PPP to produce ribose-5-phosphate (59). Recently, Louis et al. detected a higher concentration of glucose and a lower level of alanine in the plasma of lung cancer patients through nuclear magnetic resonance (NMR) metabolomics (25). These findings suggest the role of a compensatory gluconeogenesis to sustain high glucose levels in plasma to support the ongoing glycolysis in cancer cells. Here, in contrast with the rescue pathway proposed by Vincent et al., the source of glucose is the gluconeogenesis of healthy cells.

## The Role of the TCA Cycle and Oxidative Phosphorylation

In contrast with the original hypothesis of Warburg, cancer cells have functional mitochondria that act as biosynthetic hubs. Respiration, oxidative metabolism and other mitochondrial pathways are required by malignant cells for tumor growth (3, 60). An important metabolic pathway that occurs in the mitochondrial matrix is the TCA cycle or Krebs cycle. The TCA cycle is composed of biochemical reactions that oxidize fuel sources to provide ATP, support the synthesis of macromolecules and regulate the cellular redox balance. Moreover, the TCA cycle provides precursors of various amino acids. When TCA cycle intermediates, such as glucose- and glutamine-derived α-KG, are diverted for synthesis of macromolecules and ATP they need to be replaced to permit the sustained function of the TCA cycle by anaplerosis. This process is accomplished via two major pathways: glutaminolysis and carboxylation of pyruvate to OAA via pyruvate carboxylase (PC). As this review focuses on the disturbed glucose metabolism, we refer the interested reader to our recently published review that describes the role of glutamine in lung cancer (7). An important step in the TCA cycle is the conversion of isocitrate to α-KG by isocitrate dehydrogenases (IDH) and thereafter to succinate and fumarate by succinate dehydrogenase (SDH) and fumarase (FH), respectively. Mutations in genes encoding for IDH, FH, and the SCD complex lead to an altered metabolism, i.e., accumulation of TCA cycle metabolites, that enhances cell transformation by epigenetic alterations (61). Mutations in IDH1 and IDH2 are found in 1% of NSCLC and result in the conversion of α-KG to 2-hydroxyglutarate (62). This oncometabolite is considered as a competitive inhibitor of multiple dioxygenase enzymes that use α-KG as a cofactor, such as histone demethylases and TET (ten-eleven translocation) proteins resulting in DNA and histone methylation alterations and epigenetic changes altering gene expression (61). In addition, both TET2 and 2-hydroxyglutarate block differentiation in hematopoietic cells. Inactivating mutations of SDH and FH have been identified in several cancers and result in accumulation of succinate and fumarate, respectively. Succinate and fumarate are capable of inhibiting multiple α-KG dependent dioxygenases. Due to inhibition of prolyl-hydroxylases, HIF1 accumulates in SDH and FH mutant tumors and promotes metabolic rewiring of the glucose metabolism.

The voltage-dependent anion channel (VDAC1) is considered as the mitochondrial gatekeeper. The VDAC1 is the main transport channel for metabolites and its overexpression in many cancers indicates that this mitochondrial pore contributes to the metabolic phenotype of cancer cells (63). Along the regulation of the metabolic and energetic homeostasis, VDAC1 functions as a regulator of the redox balance by its capacity to transport ROS. In addition, the mitochondrial pore is involved in the process of apoptosis by interaction with inhibitors of cell death and the release of apoptotic proteins. For example, binding between VDAC1 and HK2 leads not only to a metabolic benefit but also results in the inhibition of apoptosis offering the cell not only a proliferative advantage but also protection against chemotherapy induced cell death. Downregulation of VDAC1 expression in cancer may impair the exchange of metabolites between the cytosol and the mitochondria leading to inhibition of growth and proliferation of cancer cells and their ability to evade apoptosis. The OXPHOS pathway effectively generates ATP by electron transport through several protein complexes across the mitochondrial membrane. As previously described, OXPHOS is often downregulated in hypoxic cancer tissue to limit the production of ROS by the mitochondrial respiratory chain. Warburg proposed that a decreased OXPHOS induced the enhanced glycolysis due to mitochondrial defects. This concept has been applied to all types of cancer cells without appropriate experimental evaluation. However, recently, Moreno-Sanchez described the contribution of OXPHOS in lung cancer and several other cancers. In contrast with previous assumptions, the majority of ATP in cancer cells is produced during OXPHOS (64). Indeed, studies by Hensley et al. and Davidson et al. reveal that both glycolysis and mitochondrial OXPHOS are elevated in non-small cell lung tumors (65, 66). Many other authors nowadays also support the idea that mitochondrial OXPHOS might actually be suppressed as a result of the dominating strong upregulation of the glycolysis, rather than being initially impaired as stated by Warburg. This means that OXPHOS might serve as an additional rescue energy alternative in cancer cells, in case of glycolysis inhibition (67, 68). The other way around, OXPHOS can also be preferred for energy production in normoxic conditions in order to spare glucose which can be used in an hypoxic environment.

Lactate, produced by glycolysis in both cancer cells and carcinoma-associated fibroblasts (CAFs), is converted to pyruvate and enters the mitochondria of aerobic lung cancer cells to undergo OXPHOS to generate ATP (69). This lactate shuttling, mainly via MCT1 and MCT4, is one important way how cancer tissue keeps the interplay between glycolytic and oxidative cells in balance (22). A plausible explanation might be found in the heterogeneity of lung tumors. They show to exhibit both the glycolytic and oxidative metabolic phenotype between different regions inside the same tumor (65). It seems that cancer cells of the same tumor can be divided into subgroups, often depending on their microenvironment: highly glycolytic with lower OXPHOS in hypoxic conditions and the other way around where nutrients are rather low (68). Strikingly, some lung tumors that have acquired resistance against targeted therapy also seem to switch to elevated OXPHOS activity, leaving it vulnerable for inhibition (70).

Since different types of cancer rely on the OXPHOS pathway for their development, OXPHOS inhibition is a target of several cancer therapy studies (71). For example, NSCLC tumors with LKB1 (liver kinase B) tumor suppressor mutation are shown to be sensitive to phenformin, as it shuts down oxygen consumption in these cells by inhibition of the protein complex I of the oxidative respiratory chain. Instead of reprogramming to using glycolysis for ATP generation, LKB1 mutated NSCLC cells are shown to exhibit an OXPHOS-driven phenotype (72).

## Genetic Regulation of Lung Cancer Metabolism

Lung cancer cells often harbor mutations in genes and pathways, such as the PI3K (phosphoinositide-3-kinase)-AKT-mTOR (mammalian target of rapamycin) pathway, the oncogenes RAS, c-MYC, and HIF-1 (hypoxia inducible factor), and the tumor suppressor gene TP53 (tumor protein) (73–78). These cell signaling pathways are implicated in the metabolism by securely regulating the capacity of cells to obtain access to nutrients and subsequently process these compounds.

### PI3K-AKT-mTOR Pathway

The PI3K-AKT-mTOR pathway, one of the signaling pathways most frequently altered in cancer, is an essential regulator of metabolism, coordinating the uptake and fate of glucose (74, 75, 79). The PI3K-AKT-mTOR pathway can be aberrantly activated by multiple factors including oncogenic genomic alterations in e.g., PI3K, PTEN (phosphatase and tensin homolog), AKT, TSC (tuberous sclerosis complex), LKB1, and mTOR (80). The binding of ligands, such as epidermal growth factor, to receptor tyrosine kinases, results in dimerization of the receptors which stimulates the receptor's intrinsic cytoplasmatic kinase activity, leading to auto- and transphosphorylation on tyrosine residues, which serves as docking sites of several proteins and enzymes. Recruitment of PI3K to the membrane results in the phosphorylation of the membrane compound phosphatidylinositol 4,5-bisphosphate (PIP_2_) to phosphatidylinositol 3,4,5-trisphosphate (PIP_3_). The serine/threonine kinase AKT is recruited to the plasma membrane along with PI3K-dependent kinase 1 which has been recruited and activated by PIP_3_. Phosphorylation of specific threonine and serine residues by PI3K-dependent kinase 1 and mTORC2 is essential for complete AKT activation. Once activated, AKT potentially phosphorylates many proteins which explains its broad range of downstream effects in angiogenesis, apoptosis, differentiation, and proliferation. In contrast, PTEN is a phosphatase that reduces the intracellular levels of PIP_3_ and functions as a tumor suppressor by inhibition of the AKT signaling cascade. AKT also fulfills a critical role in the uptake and metabolism of glucose by promoting the transcription of several glycolytic enzymes, such as HK, PFK1, and recruitment of GLUTs to the cell membrane (81, 82). While overexpression of nutrient transporters can help cells to harvest scarce blood-born nutrients, it has become recognized that malignant cells acquire the capacity to bypass the blood circulation and obtain nutrients by scavenging macromolecules from the microenvironment i.e., extrinsic scavenging. In contrast to autophagy or intrinsic scavenging, extrinsic scavenging can maintain survival and promote growth (83). Macropinocytosis begins with the activation of RAC1, a small GTPase, and a cell division control protein that produces ruffles that form circular cups. Closure of these cups depends on both PIP_3_ production and RAC1 inhibition. Inactivation or loss of PTEN, elevates the intracellular PIP_3_ levels which results in the stimulation of the uptake of macropinosomes by murine fibroblasts (83, 84). Furthermore, PTEN inhibition in these fibroblasts allowed them to grow even in a nutrient-depleted medium in a manner that depends on macropinocytosis. Whether other tumor types with reduced PTEN activity, such as lung cancer, use macropinocytosis to support growth, requires further research.

Downstream of PI3K and PTEN, activated AKT inhibits TSC2 via phosphorylation. Inactive TSC2 is uncapable to bind RHEB, which enables its activation of mTORC1 initiating its effect on downstream proteins that play a role in protein translation. Activation of mTOR can drive metabolic processes through the regulation of metabolic gene expression. These processes include glucose import and glycolysis via HIF-1, and the PPP (nucleotide biosynthesis and reducing equivalents for fatty acid synthesis) through sterol regulatory element-binding proteins (SREBPs).

### RAS-RAF-MEK-MAPK Pathway

The RAS family encodes four membrane-bound proteins that are involved in signal transduction underlying diverse cellular activities, such as differentiation, growth, migration, proliferation, and survival (85). Activation of RAS proteins at the cell membrane by growth factors results in the binding of effector molecules, formation of signaling complexes and initiation of a cascade of intracellular signaling pathways including the RAS-RAF-MEK-MAPK-and PI3K-AKT-mTOR pathway. RAS proteins alternate between GTP- and GDP-bound conformations, where the GTP-bound conformation represents the active state. Oncogenic mutants function by preventing hydrolysis of GTP, thereby generating highly active RAS molecules resulting in uncontrolled growth and malignant transformation. Activating (K)RAS mutations are prevalent in ~15–20% of NSCLC and 30–50% of the adenocarcinoma subtype (73). KRAS mutations are mutually exclusive to EGFR mutations and predict resistance to EGFR TKI and chemotherapy (86, 87). Another RAS effector family is PI3K, which implicates that some of the effects of RAS may be mediated through the PI3K-AKT-mTOR pathway. Indirectly, activating RAS mutations results in the upregulation of many glycolytic enzymes and transporters (55). RAS-transformed cancer cells overcome limitations of nutrients by scavenging extracellular fluid and macromolecules (e.g., albumin, extracellular matrix proteins, necrotic cell debris, …) by generating large vesicles i.e., macropinosomes. The building blocks that make up these macromolecules can be released after degradation and used for the generation of ATP and biosynthetic purposes. In analogy with KRAS-driven pancreatic cancer cells, KRAS-mutated lung cancer cells also exhibit constitutive macropinocytosis. However, *in vitro* findings show that KRAS-driven lung cancer cells degrade less albumin than isogenic lines derived from the pancreas. This observation raises the possibility that changed characteristics of the tissue of origin also control scavenging in cells with identical genomes (88). Though this intriguing result, an important caveat of this study is the *ex vivo* monitoring, which may not reflect how these cells behave within tissues. Indeed, other pathways that modulate the macropinocytic flux may be affected by both the tumor micro-environment and the mutational load. Additional studies are indispensable to ascertain whether the same KRAS-mutation leads to different amounts of macropinocytic flux in different tissue types.

### c-MYC

The MYC proto-oncogene members are targets of RAS and PI3K-AKT-mTOR signaling and critical regulators of numerous downstream pathways, such as apoptosis, differentiation, and proliferation (89). The MYC oncogene family is frequently deregulated in both NSCLC and SCLC. Activation of MYC members often occurs through amplification although excess MYC expression can also result from retroviral promotor insertion, chromosomal translocation, activation of enhancers within the MYC gene or mutations of upstream signaling pathways that enhance MYC stability (90). Concerning metabolic reprogramming, the c-MYC transcription factor promotes expression of glycolytic target genes (GLUT, HK, PFK1, and ENO) and LDH contributing directly to the Warburg effect (91, 92). MCT4, another c-MYC target extrudes lactic acid produced from glucose. It is particularly notable that c-MYC not only drives the expression of glycolytic enzymes but also favor specific mRNA splice variants, such as PKM2 over PKM1. As a consequence, c-MYC-driven accumulation of glycolytic intermediates fuels pathways that share intermediates with glycolysis, such as the PPP and the one-carbon metabolism (92). Besides, c-MYC induces expression of enzymes involved in the synthesis of nucleotide metabolism, including SHMT, which allows glycolytic carbon units to be used in the synthesis of purines and pyrimidines (92–94). Furthermore, c-MYC is also involved in the induction of pyruvate dehydrogenase kinase-1 (PDK1), an enzyme that participates in the regulation of the pyruvate dehydrogenase complex (PDH). This enzyme catalyzes the decarboxylation of pyruvate to acetyl-CoA, thereby linking glycolysis to the TCA cycle. PDK1 inhibits PDH by phosphorylation, resulting in increased conversion of pyruvate to lactate, and limiting the entry of glycolytic carbon substrates into the TCA cycle (95, 96).

### HIF-1

The transcription factor HIF is a heterodimeric complex composed of an unstable oxygen-dependent α-unit and a stable oxygen-insensitive β-unit. Under normal O_2_ conditions, the α-subunit of HIF is hydroxylated by prolyl-dehydroxylases, allowing recognition and ubiquitination by the Von Hippel Lindau ubiquitin ligase, which labels them for rapid degradation (97). In hypoxia, prolyl-dehydroxylases are inactive as they require O_2_ as an essential cofactor. In the nucleus, the stabilized HIF α-subunit dimerizes with HIF-1β and induces the transcription of many genes involved in proliferation, apoptosis, and angiogenesis (98). HIF-1 expression is absent in healthy lung tissue in contrast with cancerous lung tissue, where increased levels of HIF-1 are documented (76, 77). The significant metabolic effect of HIF-1 is to trigger the switch from OXPHOS to anaerobic glycolysis. HIF1 induces the expression of GLUT and upregulates many genes affecting glucose metabolism, such as HK, PGI, ALDO, PGK1, PDK1, ENO, PKM2, and LDH (98–100). Furthermore, HIF-1 participates in the synthesis of serine and the one-carbon metabolism by transactivation of PHGDH and SHMT, which both increase NADPH generation and defense against ROS under hypoxic conditions (101, 102).

### TP53

In lung cancer, TP53 is a commonly inactivated tumor suppressor gene. TP53 encodes a protein, p53, that prevents the accumulation of genetic damage during mitosis. In response to cellular stress, p53 induces the expression of genes that regulate cell cycle checkpoints, resulting in G1 arrest and DNA repair or apoptosis (103). Wild type TP53 inhibits transcription of glucose transporters, promotes the expression of Tumor Protein 53-Induced Glycolysis and Apoptosis Regulator (TIGAR), and inhibits the transcription of glycolytic enzymes like PGM (104). By decreasing the level of fructose-2,6-bisphosphate, TIGAR decreases the activity of PFK1, the key enzyme of glycolysis (105). Wild type TP53 supports the expression of PTEN, which inhibits the PI3K pathway, thereby suppressing glycolysis. Additionally, wild type TP53 promotes OXPHOS by activating the transcription of cytochrome c oxidase assembly protein 2 (SCO2), which is required for the assembly of the cytochrome oxidase complex of the electron transport chain. Mutations or deletions in TP53 in cancers result in the stimulation of glucose transport and glycolysis by expression of PGM and inhibition of TIGAR. Wild type TP53 also suppresses the oxidative phase of the PPP by directly binding to G6PD and repressing the enzyme activity. Cancer-associated mutations in p53 have been shown to result in loss of the ability to block G6PD activity, resulting in an increased PPP flux and glycolysis (106).

## Therapeutic Implications of Targeting the Metabolic Hallmark of Cancer

Treatment of lung cancer is moving toward the design of drugs that specifically target aberrant pathways involved in carcinogenesis (107). The increased dependence of lung cancer cells on fermentation provides a biochemical basis for the development of antineoplastic treatments that preferentially target cancer cells by pharmacological inhibition of anaerobic glycolysis. One of the advantages of metabolism-based therapeutics over gene-based therapies are the standard shifts in metabolism observed in cancers derived from many tissues. Indeed, the mechanisms underlying cancer development are incredibly complex, and genetic alterations are heterogeneous even in a specific cancer type. As a consequence, targeting a single gene is difficult and an alternative strategy is to take advantage of the fundamental difference between cancer cells and their regular counterparts. In the past decades, it has become increasingly evident that many metabolic pathways are altered in cancer cells (3, 50, 104, 108). According to Altenberg et al., glucose transporters and glycolytic enzymes are overexpressed in 24 different types of cancer, including lung cancer (19). As previously described, the disturbed glucose metabolism is driven by signal pathways and transcription factors. Inhibition of these pathways and more downstream targets, such as glucose transporters, glycolytic (iso)enzymes, or the mitochondrial pore (VDAC1), provides a tempting avenue for the development of new anti-cancer drugs. Several inhibitors (Table 1) of glycolytic enzymes and transporters are in (pre)clinical development, however only inhibitors of IDH have reached approved status. Nevertheless, there are disadvantages to a metabolism-based approach as well. Since the identical metabolic pathways are necessary for the cell division and survival of all cells, metabolism-based treatment face a major hurdle of non-specific toxicity. Immune cells, such as cytotoxic T lymphocytes, are often found in the tumor microenvironment and immune stimulation leads toward an increased demand for glucose. The glycolytic pathway does not only support the proliferation of immune cells but is also crucial for their functional activity, such as the production of cytokines and ATP (144). Therefore, activated immune cells might be expected to be vulnerable to glycolytic inhibition, resulting in immune suppression which is concerning because reactivation of the suppressed immune system has become a first line treatment in PD-L1 positive NSCLC (145, 146). A pitfall in the trials planned to test drugs targeting metabolism is the lack of knowledge of the metabolic pathways because no metabolic profiling has been performed before the initiation of therapy. Indeed, although the aerobic fermentation is the most observed phenotype, it is not a universal trait of all human tumors. In addition, due to the metabolic plasticity exhibited by cancer cells, it is not unexpected that tumor cells could develop resistance to inhibition of a specific pathway through upregulation of alternative pathways. As previously mentioned, continued functioning of the TCA cycle requires the replenishment of intermediates that are diverted for synthesis of ATP and macromolecules. The increased uptake of the anaplerotic substrate glutamine and its metabolic conversion products glutamate and α-KG contribute to the biosynthesis of all cellular constituents. Therefore, concurrent inhibition of the glutaminolysis pathway using small molecules, such as BPTES, compound 968 or CB-839 may be a valuable treatment strategy (7).

**Table 1:** Some inhibitors of glycolytic enzymes and transporters which are in (pre)clinical development.

**Target**	**Drug**	**References**	**Remark**
GLUT	Fasentin, phloretin, STF-31, WZB117	(109–112)	Preclinical models
HK	Lonidamine	(113–119)	Only one study with survival benefit
	2-deoxyglucose	(120, 121)^1^	Activation of proapoptotic pathways, probably an only role in combination with chemotherapeutic treatments
	Bromopyruvate	(122–126)	Rapid inactivation, venous irritation, lack of crossing blood-brain barrier prevents its clinical development.
			Role in the restoration of chemo susceptibility
PFKFB	3PO	(127)	Preclinical models
	PFK158	(128)	NCT02044861
GAPDH	Bromopyruvate	(124, 126, 129)	Rapid inactivation, venous irritation, lack of crossing blood-brain barrier prevents its clinical development.
			Role in the restoration of chemosusceptibility
PKM2	Shikonin	(130)	Inhibitor PKM2
			Both activators and inhibitors of PKM2 could be beneficial dependent on oxygen levels in cancer cells
LDH	FX11	(131)	Inhibition progression human lymphoma and pancreatic xenografts
	Quinoline-3-sulfonamide	(132)	Unacceptable pharmacokinetic profile preventing further investigation *in vivo* models
	Oxamate	(133)	Role in the restoration of chemosusceptibility
	GNE-140	(134)	High potency, modest permeability and a low plasma protein binding
	PSTMB	(135)	Induction of apoptosis in lung cancer cell lines
PDK	Dichloroacetate	(136)	Phase 2 trial in brain cancer NCT00540176
		(137)	Low potency, a requirement of high doses resulting in significant toxicities
			Preclinical in lung cancer NCT01029925 Terminated due to higher than expected risk/safety concerns.
	AZD7545	(138)	
MCT1	AZD3965		Currently tested in phase 1 clinical trial (NCT01791595)
IDH	Enasidenib	(139)	Approved in relapsed/refractory IDH2 mutant AML
	Ivosedinib	(140)	Approved in relapsed/refractory IDH1 mutant AML
			NCT02989857 (Phase 3 in IDH-mutant cholangiocarcinoma)
			NCT03343197 (Phase 1 in IDH-mutated glioma)
	GSK864		Preclinical, potent IDH1 inhibitor
	GSK321		Preclinical, potent IDH1 inhibitor
VDAC1	Lonidamine	(118)	Preclinical, induction of apoptosis
	SiRNA	(141–143)	Rewiring of tumor cell metabolism, reduction of cancer stem cell levels and induction of differentiation in cell lines and xenografts of glioblastoma, lung cancer and breast cancer

### Glucose Restriction and Diabetes Control

Both hyperinsulinemia and hyperglycemia are predictors of cancer incidence and worse survival in patients with various cancers as demonstrated by retrospective studies (147–150). It is unknown whether the reduction in insulin levels can affect tumors that are already present. Carbohydrate restriction and pharmacological approaches to reduce the levels of insulin may result in the development of insulin-dependent diabetes in euglycemic subjects and thus in increased glucose levels and overfeeding of tumor cells.

Recently, Ohkuma et al. published a large systematic review that confirmed the higher risk of cancer in diabetics (147). The activation of the IGFR1-IR-PI3K-AKT-mTOR pathway through hyperglycemia and hyperinsulinemia has been suggested as a cause of carcinogenesis. Indeed, binding of insulin and IGF to their receptor tyrosine kinase results in autophosphorylation of the receptors and activation of the PI3K-AKT-mTOR pathway. In addition, mTOR is negatively affected through activation of AMPK, which can also be achieved by dietary restriction (151). This previously described hyperactivation of the IGFR1-IR pathway does not occur through genetic mutations, but co-existence of cancer-associated mutations in these pathways may result in an even more pronounced promotion of growth and survival in malignant cells (152). Masur et al. showed that diabetogenic glucose concentrations compared to physiological levels resulted in different expression of genes that promote adhesion, migration, and proliferation in several cancer cell lines (153). The addition of insulin to the glucose-enriched culture medium further increased the rate of proliferation and promoted activation of the PI3K-AKT-mTOR pathway (153). It could be hypothesized that high glucose and the resulting release of insulin provides additional stimuli for neoplastic cells. However, as demonstrated by Louis et al., cancer leads to increased gluconeogenesis that is fueled by glycerol from lipolysis and alanine from rhabdomyolysis. As a consequence, higher levels of glucose are available for cancer cells, resulting in fat loss and muscle wasting, both hallmarks of cancer cachexia. As sarcopenia is related to a poor prognosis and a substantial loss in the quality of life, carbohydrate restriction has no established role in the treatment or prevention of cancer (154, 155). A switch from carbohydrate metabolism to fatty acid metabolism by diets poor in carbohydrates and rich in fats, i.e., ketogenic diets, may result in anti-cachectic effects. Based on the ability of healthy cells to use ketones as energy source, ketogenic diets have been proposed to treat glioblastomas (156). In general, the current phase I and II studies are hampered by poor accrual and compliance, and until present, no randomized controlled trials have been terminated to study the potential effects of a ketogenic diet on tumor growth and survival.

### Inhibition of Glucose Transport

Targeting GLUTs could be an efficient anticancer approach since tumor cells depend on increased utilization of glucose. This difference in glucose addiction between cancer and healthy cells provides a therapeutic window by which glucose uptake in cancer cells can be efficaciously suppressed with significantly less toxic effects in healthy cells. Inhibition of glucose importers is equivalent to the inhibition of the entire glycolytic pathway. Cancer cells will have to use other transport mechanisms, such as macropinocytosis or other metabolic fuels, such as glutamine, to compensate for the shortage of glucose. Although it is possible to acquire these compensation mechanisms, such adaptations are more complicated then bypassing the inhibition of a single enzyme in the glycolytic pathway (88). Based on physiological requirements for glucose, different isoforms of GLUTs are expressed in various cell types. In cancer, GLUT1 and GLUT3 are the most relevant transporters. GLUT1 is a fundamental transporter expressed in almost all cell types, and its upregulation in cancer cells is well-documented (17, 19). Unlike GLUT1, GLUT3 is expressed primarily in tissues with high energy demand to supplement GLUT1. Several inhibitors of glucose transporters, such as fasentin, phloretin, STF-31, and WZB117 have already been discovered, and experiments with preclinical models demonstrated their anticancer effects (109–112). For example, as demonstrated by Liu et al., the treatment of lung cancer cells with WZB117 did not only result in decreased levels of GLUT1 protein but also in a decline in the concentration of intracellular ATP and glycolytic enzymes (112). Furthermore, these authors demonstrated that intraperitoneal injection of WZB117 resulted in a significant reduction of tumor volume *in vivo* in a nude mouse xenograft model.

Research by Wood et al. documented that fasentin not only partially inhibited glucose transport but also broke down the resistance of caspase activation which usually is seen in cells that are resistant to antineoplastic treatment (110). Despite these exciting findings, inhibitors of GLUTs have not yet entered clinical trials.

### Inhibition of Hexokinase (HK)

In addition to the inhibition of glucose transport, the glycolytic pathway can be inhibited at the enzymatic level. Lonidamine is a selective inhibitor of the soluble and mitochondrial-bound HK2 iso-enzyme, which is present in malignant cells but not in healthy cells and is effective in the treatment of diverse cancer cells (113–115). However, the combination of lonidamine and chemotherapy did not improve the time to progression in breast cancer patients, and its hepatoxicity resulted in early termination of clinical trials (116, 117). The inhibition of HK2 by lonidamine leads to decreased glucose phosphorylation, which results in lower concentrations of glucose-6-phosphate and as a consequence, results in a reduction of glycolytic intermediates and the PPP. Furthermore, in cancer cells, HK2 associates with the voltage-dependent anion channel (VDAC1), located on the outer mitochondrial membrane, to protect malignant cells from mitochondrial membrane permeabilization. Ravagnan et al. showed that supernatants of mitochondria that were processed with lonidamine contain several factors, including cytochrome C, capable of inducing apoptosis (118). These findings indicate that lonidamine acts through the opening of the mitochondrial permeability transition pore. Indeed, targeting VDAC1 by small molecules or VDAC1-based peptides that interfere with anti-apoptotic proteins results in the induction of apoptosis, making VDAC1 an interesting target to overcome resistance to chemotherapy. Furthermore, strategies using specific small interfering RNA (siRNA) in glioblastoma cells lines and xenografts resulted in a rewiring of tumor cell metabolism, a reduction of cancer stem cell levels and induced differentiation into neuron- and astrocyt-like like cells (141). Similar results, regardless of cell origin and genetic mutational burden, were obtained in lung cancer and breast cancer cell lines and in mouse xenografts (142, 143). As demonstrated by Arif et al., VDAC1 depletion resulted in depletion of transcription factors coordinating cell metabolism, such as c-MYC and HIF-1, finally leading to differentiation, independent of cell type and genetic alterations (142). Therefore, VDAC1 is an interesting target for treating various cancers.

Encouraging data of phase 1 and 2 trials have led to testing lonidamine in several phase 3 trials in several cancers including lung cancer Unfortunately, these results were rather disappointing as only one study detected a statistically significant higher response rate and better survival in patients treated with lonidamine-containing regimens. The glucose analog 2-deoxyglucose, another inhibitor of HK2, demonstrated promising effects in preclinical models (157). Despite the results, its success as a single glycolysis inhibitor has become controversial as the drug activates multiple pro-survival pathways in cancer cells and studies in prostate cancer documented insignificant effects on tumor growth^1^ (120). Combination therapy of paclitaxel and 2-deoxyglucose in a NSCLC xenograft model resulted in a remarkable reduction in tumor growth than when compared with either agent alone (121). This observation presents a rationale for the initiation of clinical trials using chemotherapy in combination with 2-deoxyglucose, in order to increase their clinical effectiveness.

### Inhibition of Phosphofructokinase Isoforms (PFK)

As previously described, the conversion of fructose-6-phosphate to fructose-1,6-bisphosphate by PFK1 is the committed rate-limiting step of glycolysis. Fructose-2,6-bisphosphate is a potent activator of PFK1. The concentration of fructose-2,6-bisphosphate is determined by a family of bifunctional enzymes PFK-2/FBP (PFKFB) which consists of four iso-enzymes. The high kinase/phosphatase ratio of the iso-enzyme encoded by the PFKFB3 gene, results in sustained high glycolytic rates. As in colon cancer, loss of PTEN, stabilization of HIF-1, and activation of RAS in lung cancer cells, converge to increase the activity of PFKFB3. The small-molecule inhibitor 3PO inhibits the PFKFB3 iso-enzyme through competition with fructose-6-phosphate without inhibition of PFK1 activity. *In vitro*, 3PO attenuates the proliferation of several human cancer cells and exhibits selective cytostatic activity to RAS-mutated epithelial lung cancer cell lines relative to their healthy counterparts (127). *In vivo*, the administration of 3PO reduces growth of lung adenocarcinoma cells. The optimization of this class led to a more potent inhibitor of PFKFB3, i.e., PFK158. *In vitro*, PFK158 results in a decreased uptake of glucose and the release of lactate as well as induction of apoptosis in gynecologic cancer cell lines (128). Furthermore, PKF158 treatment sensitizes chemoresistant cells and induces cell death. These findings indicate that chemotherapy in combination with PFK158 may have a role in the treatment of chemoresistant cancer. Safety and toxicity studies in animals have demonstrated that PFK158 is well-tolerated with a good therapeutic index, lending further support for a phase 1 clinical trial in patients with metastatic solid malignancies (NCT02044861).

### Inhibition of GAPDH

The glycolytic enzyme GAPDH plays a critical role in the cellular redox balance by the generation of NADH, which is involved in the regulation of ROS and in biosynthetic processes of macromolecules. Apart from its glycolytic function, tumor-specific roles of GAPDH include chemoresistance, metastatic potential, protection of cancer cells from apoptosis, and cell cycle regulation (158–160). Given the central role of GAPDH, its inhibition triggers a cascade that may lead to cell death. Under normal conditions, degradation of accumulated GAP and DHAP results in the formation of the cytotoxic metabolite methylglyoxal, which enters the glyoxalase system to undergo detoxification. However, in the presence of oxidative stress and glutathione depletion, the glyoxalase system fails to detoxify the cytotoxic metabolite resulting in apoptosis (161). Several GAPDH inhibitors have been tested in cell cultures and animal models for their efficacy (162). However, the ubiquitous nature of GAPDH and the resulting systemic toxicity needs to be addressed in clinical trials. A promising GAPDH inhibitor is the pyruvate analog 3-bromopyruvate. Bromopyruvate is a powerful anti-cancer agent that not only interferes with the process of glycolysis but also impacts the TCA- and folate cycle (122, 163). Unfortunately, the molecule faces many biochemical and practical problems, such as rapid inactivation by the thiol groups of e.g., glutathione and venous irritation during infusion (164). Lack of early tumor response, the resistance of cells rich in glutathione, the lack of crossing the blood-brain barrier, and the phenomenon of enhanced permeability and retention prevents the approval of 3-bromopyruvate in clinical trials. Notwithstanding the induction of apoptosis in breast cancer cell lines, bromopyruvate was observed to trigger autophagy, which increased resistance to bromopyruvate treatment (123, 129). In colon cancer, bromopyruvate treatment rendered resistant cells susceptible to 5-fluorouracil and oxaliplatin (124). Malignant cells, treated with bromopyruvate, were observed to have a larger uptake of chemotherapeutic drugs resulting in a restoration of susceptibility to these drugs. Overexpression of drug-expelling ATP-binding cassette transporters (ABC) prevents accumulation of chemotherapeutic drugs into cancer cells, eventually leading to drug resistance. Since these transporters are dependent on ATP production through enhanced glycolysis, inhibition of the glycolytic pathway with bromopyruvate may restore the susceptibility of malignant cells to chemotherapy.

### Pyruvate Kinase (PK): Inhibitors or Activators?

The discovery that the expression of PKM2 results in a growth advantage for malignant cells raised the hypothesis that the enzyme could be an interesting target for cancer treatment. The inhibition of PKM2 may result in the accumulation of glycolytic intermediates that feed biosynthetic pathways resulting in tumor proliferation. As demonstrated by Anastasiou et al., oxidative stress results in the oxidation of PKM thereby suppressing its activity and supporting the diversion of glycolytic intermediates into the PPP resulting in the generation of NADPH and restoration of the redox balance (165). Activators of PKM2 could be interesting cancer drugs, mainly when administered in combination with treatments that disrupt the cellular redox balance, such as radiotherapy and chemotherapeutics. In contrast, other investigators demonstrated that inhibition of PKM2 increases cell death in mouse xenograft models (166). This discrepancy may result from different cellular responses to variable degrees of hypoxia (167). Mild hypoxia results in the production of hydrogen peroxide, which ultimately promotes signaling pathways that are critical for the response to hypoxia. In this setting, oxidation of PKM2 leads to inactivation of the glycolytic flux and increased flow through the PPP. As a result, the production of NADPH prevents the accumulation of ROS and oxidative damage. During severe hypoxia, the O_2_ supply to the electron transport chain becomes compromised, resulting in a reduction of mitochondrial ATP production and hydrogen peroxide. As a consequence, cancer cells depend on the PK activity for the production of ATP. In conclusion, depending on the degree of hypoxia, both PKM2 activators and inhibitors could be beneficial. Indeed, in severely hypoxic cells PKM2 inhibitors may prevent ATP production, whereas PKM activators may result in oxidative damage in cells with moderate O_2_ levels. Shikonin is a potent and specific inhibitor of PKM2. Incubation of lung cancer cells with shikonin resulted in a reduced glycolytic rate as manifested by decreased glucose consumption and lactate production (130).

### Inhibition of Pyruvate Dehydrogenase Kinase (PDK)

PDKs and PDH are mitochondrial enzymes that determine the proportion between the Warburg effect and aerobic respiration (168). As overexpression of PDKs has been detected in several human cancer samples and has been associated with a dismal prognosis in several other cancers, new drugs that inhibit PDKs may be used to treat a variety of cancers and may provide a new kind of antineoplastic class (96). In addition, the low expression of PDK in normal tissue may spare healthy cells and adverse effects may be minimal. Several PDK inhibitors have been reported, although their clinical efficacy needs confirmation. Dichloroacetate (DCA) has been identified as an activator of PDH by inhibition of PDK activity and has successfully entered into phase 2 trials in treating brain tumor patients (136). The consequences of DCA on lung cancer cells and animal models were explored in detail by Bonnet et al. who demonstrated that administration of DCA resulted in a shift from glycolysis to OXPHOS (137). Furthermore, this shift in metabolism led to higher levels of ROS and a decreased mitochondrial membrane potential in lung and several other malignancies without any effect on standard cell lines. The activation of the mitochondrial function resulted in apoptosis due to the efflux of pro-apoptotic mediators from the mitochondria. Despite these encouraging results, the application of DCA in the treatment of cancer is plagued by its low potency and the need for high dosages to exhibit therapeutic effects, resulting in toxicities, such as peripheral neurological toxicity (169). Due to high risk/safety concerns, the NCT01029925 trial investigating the response rate of DCA in patients with recurrent and advanced NSCLC was closed prematurely. Therefore, clinical trials with more potent and selective PDKs inhibitors, such as AZD7545 are of significant importance (138).

### Inhibition of Lactate Dehydrogenase a (LDH-A)

LDH-A has an essential role in perpetuating a high rate of glycolysis by the regeneration of NAD^+^ making it a potential therapeutic target. Inhibition of LDH-A by the small molecule inhibitor FX11 increased non-productive mitochondrial respiration, leading to reduced ATP levels, increased O_2_ consumption, ROS production, and cell death. In addition, the molecule inhibited the progression of lymphoma and other cancer xenografts (131). In combination with FK866, another metabolic inhibitor that inhibits NAD^+^ synthesis, FX11 can induce lymphoma regression. Quinoline 3-sulfonamide, another LDH-A inhibitor, has been studied in multiple cancer cell lines by Billiard et al. (132). LDH-A inhibition resulted in increased intracellular concentrations of glycolytic and TCA cycle intermediates, consistent with blockage of glycolysis and enhanced TCA cycle activity, respectively. However, the unacceptable pharmacokinetic profile, i.e., the low *in vivo* clearance and the low oral bioavailability, prevents further use *in vivo*. To improve the cellular potency of LDH inhibitors, structure based designs, such as substitution of the hydroxylactam core, were utilized to create a novel series of LDH-A inhibitors. This strategy resulted in the discovery of GNE-140, a molecule that inhibits proliferation in several cancer cell lines and mice. The combination of high potency, modest permeability and a low plasma protein binding makes it a promising metabolic drug (134). More recently, Kim et al. demonstrated that the inhibitory concentration of PSTMB was significantly lower than that of other LDH-A inhibitors which may result in less toxicity (135). These authors demonstrated that PSTMB induces apoptosis in lung cancer cell lines, through induction of ROS production. In breast cancer, it was demonstrated that LDH-A plays a vital role in taxol resistance. Treatment of breast cancer cell lines with the LDH-A inhibitor oxamate and taxol resulted in a synergistic inhibitory effect on taxol resistant cancer cells by promoting apoptosis in these cells (133). This result provides evidence for the future development and use of metabolic therapies to overcome chemoresistance.

### Monocarboxylate Transport Inhibitors

Depending upon the isoform of MCT, lactate could be imported (MCT1) or exported (MCT4). Intracellular trapping of lactate will result in intracellular acidification, causing cell death. Recent studies with AZD3965, a potent, selective inhibitor of the MCT1 have demonstrated that the drug inhibits the transport of lactate and cell growth in cancer cells. The drug is currently tested in a phase 1 clinical trial that enrolls patients with advanced solid tumors or lymphoma that are refractory to conventional treatment or for which no conventional therapy exists. In addition, the disruption of lactate/H^+^ symporters has also been studied via genetic tools. Marchiq et al. studied the effect of knocking out the BASIGIN (BSG) and MCT4 genes on the metabolism of colon adenocarcinoma and glioblastoma cells (170). In their study, the authors found a strong reduction of the rate of glycolysis as expected. However, upon inhibition of MCT1 by the MCT1 inhibitor AR-C155858, the cells O_2_ consumption increased, thus indicating a rapid shift from glycolysis to OXPHOS. The authors went one step further and showed that the disruption of MCT4 and BSG sensitized the glycolytic tumor cells to phenformin, an inhibitor of mitochondrial complex I. Due to the rapid decrease in cellular ATP by disrupting both glycolysis as well as OXPHOS, cell death by “metabolic catastrophe” was observed. This observation confirmed their larger dependency on OXPHOS following the disruption of glycolysis. Similar shifts toward OXPHOS were later reported in cancer cells following disruption of glucose-6-phosphate isomerase and LDHs as covered in a mini-review by Ždralević et al. (171).

### Inhibition of Mutant Isocitrate Dehydrogenase

As mentioned before, mutations in IDH iso-enzymes result in the production of the oncometabolite 2-hydroxyglutarate, which has been linked to the interference with metabolic and epigenetic alterations responsible for cellular differentiation. Recently, the IDH1 inhibitor enasidenib, and IDH2 inhibitor ivosidenib, were approved in the treatment of patients with acute myeloid leukemia (AML) (172). GSK864 and GSK321 are promising potent inhibitors of IDH1 but have not yet entered clinical trials. Existing clinical and preclinical data in hematologic and solid tumors and the potential limitations of treatment were recently discussed by Golub et al. (172).

## Conclusions

Metabolic instability caused by environmental influences or perturbations in certain enzymes and substrates may result in mutations in oncogenes and tumor suppressor genes, leading to activation or inhibition of signaling pathways and transcriptional networks which account for the metabolic reprogramming observed in cancer cells. These metabolic adaptations are mandatory for the requirements of rapidly dividing cells: a rapid ATP generation to maintain energy status, an increased biosynthesis of biomolecules and the maintenance of the cellular redox balance. The metabolic phenotype of lung cancer cells is characterized by increased glucose uptake and glycolytic activity. However, new insights reveal the importance of other glucose-related pathways, such as gluconeogenesis, the TCA cycle and OXPHOS. Specific variations in the metabolism of cancer depend not only on the genetic alterations but also on environmental factors, such as vascularization and the supply of oxygen and nutrients. Targeting the metabolic differences between cancer and healthy cells may turn into a novel, promising anticancer strategy. Several recent studies have focused on targeting the cellular metabolic pathways in cancer cells. However, pharmacologic studies are primarily carried out using cell lines or xenograft models. To avoid the same types of toxicity that plague the current chemotherapeutic regimens, the toxic effects of inhibiting glycolytic enzymes in healthy cells needs further investigation. Besides, due to the metabolic plasticity exhibited by cancer cells, cancer cells could develop resistance to inhibition of a particular pathway through upregulation of alternative pathways, such as glutaminolysis and OXPHOS or through interaction with neighboring cells that may also provide precursors for their metabolic needs.

## Author Contributions

KV: original draft. G-JG, LM, MT, ED, J-PN, WG, and PA: review and editing.

### Conflict of Interest

The authors declare that the research was conducted in the absence of any commercial or financial relationships that could be construed as a potential conflict of interest.

## Footnotes

^1^https://clinicaltrials.gov/ct2/show/NCT00633087
